# Physicochemical, Antioxidant Characteristics and Sensory Evaluation of Functional Pro‐Biogenic Ice Cream

**DOI:** 10.1002/fsn3.4619

**Published:** 2024-12-01

**Authors:** Mehri Soodbar, Naheed Mojgani, Mohammad Reza Sanjabi, Saeed Mirdamadi, Mostafa Soltani

**Affiliations:** ^1^ Department of Food Science and Technology, Faculty of Pharmacy, Tehran Medical Sciences Islamic Azad University Tehran Iran; ^2^ Biotechnology Department Razi Vaccine & Serum Research Institute‐Agriculture Research Education and Extension Organization (AREEO) Karaj Iran; ^3^ Agriculture Research Institute Iranian Research Organization for Science and Technology (IROST) Tehran Iran; ^4^ Department of Biotechnology Iranian Research Organization for Science and Technology (IROST) Tehran Iran; ^5^ Department of Food Science and Technology, Faculty of Pharmacy, Tehran Medical Science Islamic Azad University Tehran Iran

**Keywords:** antioxidant activity, functional ice cream, pro‐biogenic, probiotic, propolis

## Abstract

Pro‐biogenic is a recent terminology widely used for products that combine biogenic materials and probiotics which has made progressive improvement in a new era of research on functional foods. This study aimed to develop functional ice cream with *Bacillus coagulans* and propolis extract (PE) as a biogenic part to develop ice cream's physiochemical and antioxidant characteristics. Five probiotic ice cream samples were prepared using different levels of PE powder (0%, 0.2%, 0.4%, 0.6%, and 0.8% w/w), and the physicochemical, total phenol content (TPC), antioxidant and sensory properties, and probiotic survival of the samples were examined. The study found that PE levels did not significantly impact fat, protein, carbohydrate, and ash content, overrun, melting rate, and adhesiveness of probiotic ice cream, but increased dry matter, apparent viscosity, and hardness. Adding PE to freeze‐storage samples significantly (*p* < 0.05) reduced pH and improved TPC and antioxidant activity. The prepared ice cream containing probiotic bacteria and PE extracts, despite their darker and yellower color, were acceptable based on sensory evaluation. Furthermore, the survival of probiotic bacteria in the ice cream, with different levels of PE appeared to be in acceptable limits (10^7^ CFU/g). The findings of the research indicated that the pro‐biogenic ice cream has good functionality and incorporating a PE aside probiotic could improve physiochemical and antioxidant characteristics which can be used as a value‐added ingredient in the formulation of functional pro‐biogenic ice creams.

## Graphical abstract

Developed functional ice cream with *Bacillus coagulans* and propolis extract (PE). PE increased viscosity, hardness total phenol content, antioxidant activity, and probiotic survival of ice cream. Sensory evaluation deemed all probiotic ice cream samples acceptable despite altered color profiles. PE is suggested for enhancing functional properties and probiotic survival in ice cream formulations.

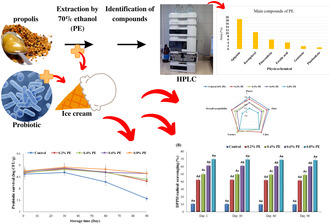

## 1 Introduction

The food industry is diligently striving to create stable, innovative, and nutritious diets to meet the growing consumer demand (Gunes‐Bayir et al. 2022). Functional foods are characterized as foods that deliver vital nutrients and enhance the overall health of consumers. In recent years, studies have associated the use of food additives with a rise in health‐related challenges. Consequently, consumers have shifted their focus towards organic, natural, and healthy food options that are free from artificial additives (İncili et al. 2025; Luvián‐Morales et al. 2022). Researchers have been investigating bioactive compounds, including antioxidants, probiotics, prebiotics, phytochemicals, oligosaccharides, proteins, dietary fibers, and choline, for their application in the production of functional foods (Anil et al. 2022; Guo et al. 2024). Recent interest has been focused on the expansion of probiotics and functional foods, which incorporate live microbial strains along with specific growth substrates (Palanivelu et al. 2022). Probiotics are defined as viable microorganisms that have a beneficial impact on the host by modulating and improving its intestinal microbial balance (Abedinia et al. 2021). There are numerous documents discussing the health‐promoting properties of probiotics. These include their ability to inhibit the growth of foodborne pathogens and toxic bacteria in the digestive tract, control cholesterol levels, have anti‐carcinogenic effects, produce bacteriocin and acid, improve the immune system, and produce β‐galactosidase with lactose hydrolyzing activity (Liu et al. 2024; Shahein et al. 2022). These properties make them suitable for people suffering from lactose intolerance (Vera‐Santander et al. 2023).

The main bacteria used as probiotics include some species of the *Lactobacillus*, *Bifidobacteria, Bacillus, Pediococcus*, *Streptococcus*, and *Enterococcus* genera (Mojgani et al. 2020). Among probiotic bacillus species, *B. coagulans* are spore‐forming, lactic acid‐producing, facultative anaerobic bacteria that are widely recognized for their stability in harsh conditions of food processing and digestion (such as heat, pressure, and acid) (Konuray and Erginkaya 2018). Several *B. coagulans* strains have demonstrated therapeutic benefits in the gastrointestinal tract and colon and have shown potential role in the treatment of gastrointestinal tract (GIT) disorders, such as irritable bowel syndrome (IBS), antibiotic‐associated diarrhea, inflammatory bowel disease, and colorectal cancer (Mazhar et al. 2024).

Propolis is a resinous product and a wax‐like paste that worker bees gather from plants, buds, and flowers of birch, oak, pine, and willow trees (Mountford‐McAuley, Prior, and Clavijo McCormick 2023). This biologically active bee product is a valuable source of biologically active materials used in the food industry as an additive (Bankova, Trusheva, and Popova 2021). A number of factors including, the harvest season, botanical and geographical origins, harvesting method, as well as post‐harvesting processing of raw propolis, have significant effects on the values of active compounds in propolis (Maldonado et al. 2020; Rivero‐Cruz et al. 2020). Some of the most important chemical active components in propolis are flavonoids, flavones, flavanones, flavanols, derivation of benzoic acid, cinnamic acid, cinnamyl alcohol, terpene, sesquiterpene, diterpene, triterpene alcohol, etc. (Irigoiti et al. 2021). Propolis has a relatively pleasant aroma and a color range from yellow to dark brown, with variations based on the region and types of plants present (de Almeida‐Junior et al. 2023). A number of studies have indicated the antiviral, antibacterial, anti‐inflammatory, anticancer, antioxidant, antifungal, hepatoprotective, antiallergic, and immunomodulatory properties of different propolis. Owing to these known benefits propolis has been widely considered for the development of functional food products (Pobiega, Kraśniewska, and Gniewosz 2019; Zulhendri et al. 2022). Dairy products serve as the primary carriers of probiotics. In this context, ice cream is a widely consumed dairy product (Mohammed et al. 2022) that has several beneficial components, including proteins, bioavailable minerals, oligosaccharides, organic acids, and bioactive substances (Yosefiyan et al. 2024). These elements contribute to significant health benefits, making dairy products an excellent medium for the delivery of probiotics (Elkot et al. 2022; Xiong et al. 2022). While studies have indicated that the addition of *B. coagulans* did not exhibit an adverse effect on the sensory and nutritional characteristics of the products. The growing interest of consumers in therapeutic products has led to the incorporation of probiotic cultures in ice cream, thus resulting in dietetic ice cream (Kobus‐Cisowska et al. 2019).

The objective of the present research was to analyze the effects of ice cream enriched by utilizing probiotic bacteria in conjunction with propolis extract. The synergistic effects of these two functional ingredients with proven health benefits could lead to development of a new health‐promoting food product that could have wide application. During studies, the impact of varying concentrations of propolis extract on the physicochemical characteristics, antioxidant activity, sensory attributes, and viability of probiotic bacteria in the ice cream was evaluated.

## 2 Material and Methods

### 2.1 Materials

Propolis samples from *Apis mellifera* were collected from native beekeepers in the region of Zaraqan (Hamadan province, Iran) at the end of July 2019. The samples were stored in the dark at −70°C before any analysis. Pasteurized cow milk, skimmed milk powder, and cream (30% fat) were obtained from Pegah Tehran, (Iran). White sugar and salep stabilizer were obtained from the local market in Tehran, Iran. The emulsifier, consisting of a blend of fatty acid mono‐ and di‐glycerides, was acquired from Sigma‐Aldrich (St. Louis, MO, USA). The probiotic bacterial strain, *Bacillus coagulans* BC‐A10 was obtained from a local Probiotic manufacturer (Biorun.co.ir, Karaj), Iran. The mentioned probiotic bacteria (1%) was cultured in Tryptic Soy Broth (TSB; Becton, Dickinson and Company, Berkshire, England) with 1% yeast extract (TSBY), and incubated at 37°C for 24 h under aerobic conditions. All chemicals used in study were purchased from Sigma Chemical Co. (St. Louis, MO, USA).

### 2.2 Preparation of Propolis Extract (PE)

Extraction of PE was conducted using ethanol as a solvent in conjunction with the ultrasound method. Ten grams of raw propolis was mixed with 100 mL of 70% ethanol, and subsequently placed in a water bath at 45°C overnight. The suspension was then subjected to an ultrasonic bath with a frequency of 20 kHz for 30 min. The resulting suspensions were filtered using Whatman filter paper (No. 41). To enhance extraction efficiency, the obtained extracts were centrifuged for 10 min at 4000 rpm. This step was repeated twice on the residual sample. The supernatant was then dried and collected in a rotary evaporator under reduced pressure. The prepared propolis extract (PE) was finally dried using a freeze dryer (ZIRBUS VACO 5, Germany) and stored in the dark at −20°C until further use (Gargouri et al. 2019).

### 2.3 Physicochemical Analysis of Propolis

#### 2.3.1 Total Phenolic Contents (TPC)

The total phenolic content (TPC) of the plant extract (PE) was assessed utilizing the Folin‐Ciocalteau method. An aliquot of the extract (0.2 mL) was combined with distilled water (1.5 mL) and the Folin‐Ciocalteau reagent (0.4 mL). Following a 5‐min incubation period, 20% (w/v) sodium carbonate solution (0.6 mL) was added and the mixture allowed to stand in dark at room temperature for 120 min. The optical density was measured at 760 nm against a blank. The TPC was calculated using a Gallic acid calibration curve ranging from 25 to 300 μg/mL, with the results expressed as milligrams of Gallic acid equivalents (GAE) per 100 g of extract (Azarashkan et al. 2022).

#### 2.3.2 Total Flavonoid Content (TFC)

TFC of PE was measured using the aluminum chloride calorimetric method, and quercetin (QE) as standard (5 to 250 μg/mL). PE was mixed with 0.5 mL of aluminum chloride (10%) and 0.5 mL of potassium acetate (1 M). Following incubation of the mixture in the dark for 30 min at room temperature, its absorbance was recorded at 510 nm with a spectrophotometer. The TFC of the PE was expressed as mg QE per 100 g of extract (Shi et al. 2022; Syed Salleh et al. 2021).

#### 2.3.3 Antioxidant Assay

To determine the antioxidant activity of PE, 0.3 mL of extract was mixed with methanol (1.2 mL) and DPPH methanol solution (1.5 mL). The mixture was then incubated for 30 min at room temperature, and its absorbance was recorded at 517 nm with spectrophotometer. The DPPH radical scavenging of the PE was calculated through the following equation (Esmaeili et al. 2024):

$$\text{DPPH radical scavenging}\;\left(\%\right)=\frac{\text{Absorbance of sample}-\text{Absorbance of blank}}{\text{Absorbance of blank}}\times 100$$

#### 2.3.4 Identification of Phytochemical Compounds

The phytochemical compounds in PE were identified utilizing RP‐HPLC (Yung Lin 9100, South Korea) with an RP‐18 column (250 × 4 mm, particle size 5 μm). A sample of PE (10 mg) was dissolved in 1 mL of 80% ethanol and subsequently filtered through a 0.2 μm nylon membrane. Following this, a 10 μL aliquot of the solution was injected into the system, with detection performed at a wavelength of 280 nm. The mobile phase for this analysis comprised (A) formic acid (0.1%) in water and (B) formic acid (0.1%) in acetonitrile. The solvent gradient commenced with 80% A and 20% B, progressing to 30% B over 10 min, 40% B at 40 min, 60% B at 60 min, and 90% B at 80 min, before reverting to the initial conditions (Gargouri et al. 2019).

### 2.4 Preparation of Probiotic Ice Cream

The probiotic inoculum was prepared by culturing *B. coagulans* (1%) in TSBY broth medium and incubated at 37°C for 24 h. The overnight culture broth was centrifuged and the collected cell pellets washed twice with sterile peptone water and cell concentrations determined in CFU/mL. Probiotic ice cream was prepared as described by Haghani et al. (2021). Initially, the cow's milk fat (5 kg) was adjusted to a concentration of 6% by incorporating cream. The milk was then portioned into five segments of one kilogram each, to which skim milk powder (5%), sugar (15%), stabilizer (0.75%), and emulsifier (0.25%) were added. The resulting mixtures underwent pasteurization for 25 s at 85°C, followed by rapid cooling to 2°C. Subsequently, *B. coagulans* was dissolved in sterilized milk to achieve approximately 10^9^ CFU/mL of bacterial cells, which was then introduced into the ice cream mixtures at a concentration of 1% (v/v). Following this, varying concentrations of PE (0%, 0.2%, 0.4%, 0.6%, and 0.8%) were incorporated into the ice creams. The whipping and freezing process (final temperature of −10°C) was conducted using an ice cream maker (Ninja, America) for 2 h, after which the ice cream samples were packed in sterile plastic cups with caps and hardened for 24 h at −23°C and subsequently stored for 90 days at −18°C.

### 2.5 Survival of Probiotic Bacteria in Prepared Ice Creams

The *B. coagulans* viable count was determined in ice cream samples on day 1, 30, 60, and 90 of freezing storage. At the mentioned time intervals, approximately 1 g of ice cream samples were diluted in 9 mL of peptone water and homogenized for I min on vortex. Serial dilutions of the samples from different were plated on Nutrient Yeast Extract Salt medium (NYSM agar; Merck, Darmstadt, Germany). The plates were incubated anaerobically for 48 h at 37°C (Janipour et al. 2023). The survival rate was estimated by enumerating the colonies appearing on the respective plates and mentioned in log CFU/g.

### 2.6 Physicochemical Properties of Prepared Ice Creams

Chemical composition of ice cream samples including fat (Gerber method), protein (Kjeldahl method), carbohydrate, ash (electric furnace) and dry matter (gravimetric method), as well as their pH and acidity (based on lactic acid) (titration method), were estimated using the AOAC methods (AOAC 2005). The apparent viscosity of ice creams was measured at 2.5 rev/min using a Brookfield Viscometer (USA) after 20 h of keeping the samples at room temperature. The overrun percentage of ice cream samples was obtained using the following equation (Makouie et al. 2021):

$$\text{Overrun}\;\left(\%\right)=\frac{\text{weight of}\;\mathrm{mix}-\text{weight of same volume of}\;\mathrm{ice}\;\text{cream}}{\text{Weight of same volume of}\;\mathrm{ice}\;\text{cream}}\times 100$$

To determine the melting rate of ice creams, first, each of the samples (50 g) was placed on the wire mesh screen at 25°C ± 1°C for 80 min (at room temperature) to melt, and then the weight of the melted ice cream was recorded and the melting rate was obtained through the following equation (Hanafi, Kamaruding, and Shaharuddin 2022):

$$\text{Melting rate}\;\left(\%\right)=\frac{\text{weight of melted}\;\mathrm{ice}\;\text{cream}}{\text{Initial weight of}\;\mathrm{ice}\;\text{cream}}\times 100$$

The color indices of the ice cream samples, including L* (brightness), a* (redness‐greenness), and b* (yellowness‐blueness), were determined using a Minolta colorimeter (Japan), and the following equation was used to calculate the total color differences (Δ*E*) (Góral et al. 2018):

$$\Delta E=\sqrt{{\left(\Delta L\right)}^{2}+{\left(\Delta a\right)}^{2}+{\left(\Delta b\right)}^{2}}$$

A 50 mL plastic falcon was filled with 30 mL of ice cream, which was then kept at −20°C for 24 h prior to the texture study. Prior to the measurement, the frozen samples were kept at 25°C for two minutes. Six measurements were made on each ice cream sample using a cylindrical stainless steel probe with a 20 mm diameter that was connected to a 500 N load cell. With a 3‐s pause in between cycles, the samples were compressed twice to 50% of their initial height at an overhead speed of 90 mm per minute. At 25°C, the trials were carried out. The maximum compression force during penetration was used to compute hardness. The negative force area of the first was found to be adhesiveness (Akalın et al. 2018).

### 2.7 Measurement of TPC and Antioxidant Activity of Prepared Ice Creams

The extracts of ice cream samples for determining TPC and antioxidant assay was performed with slight modifications in the method described earlier by Öztürk‐Yalçın, Ürkek, and Şengül (2024). Approximately 50 g of ice cream samples were added to 500 mg/L methanol solution (25 mL) and the obtained suspensions were filtered through membrane filter (0.45 μm).

### 2.8 Sensory Evaluation of Probiotic Ice Creams

To evaluation the sensory properties or probiotic ice cream samples, including flavor, odor, color, texture and overall acceptability was performed according to National Standards (ISO‐IRI 4937). A panel of 20 untrained panelists (10 women and 10 men in the age range of 20 to 40 years) evaluated the ice cream samples based on 5‐point hedonic scale (5: very good, and 1: very bad). Score 3 was considered as the minimum acceptable score.

### 2.9 Statistical Analysis

All the obtained data were analyzed statistically using one‐way analysis of variance (ANOVA) and Duncan multi‐range test to identify the significance difference between samples at *α* = 0.05 using SPSS software version 22.0 (IBM Corp., New York).

## 3 Results and Discussion

### 3.1 Identification of Phytochemical Compounds, TPC, TFC and Antioxidant Activity of PE

Propolis is an additive rich in phytochemical compounds such as flavonoids, carboxylic acids, terpenoids, quercetins, and steroids, which exhibit significant biological activities, including anti‐inflammatory, antibacterial, and antioxidant effects. The propolis used in this research was collected from the Zaraghan region of Iran, and its TPC, TFC, and DPPH radical scavenging activity were 4136.48 mg GAE/g, 2068.24 mg QE/g, and 75.08%, respectively. Consistent with these results, do Nascimento et al. (2018) also reported that the TPC of PE (9.39–12.22 mg GAE/mL) was higher than its TFC (2.83–7.05 mg QE/mL). While in contrast, Pratami et al. (2018) reported TFC values of their PE to be higher than TPC values. The higher TFC in propolis extracts compared to TPC is likely due to a combination of factors, including the prevalence of flavonoids within the extract, efficient extraction of flavonoids, the biological role of flavonoids in plant defense, and their strong correlation with antioxidant activity. In this study, the DPPH radical scavenging activity of PE (92.3%–97.1% at a concentration of 50 μg/mL) was higher than the value obtained in the present study.

As presented in Table 1, the phytochemical compounds in the PE were analyzed by RP‐HPLC. A total of 12 compounds were identified in the examined extract of propolis, with the most significant quantities attributed to apigenin (18.9%), kaempferol (10.6%), pinocembrin (5.9%), ferulic acid (4.1%), genistein (1.8%), and pinobanksin (1.0%). Other compounds were detected in considerably lower concentrations. Gargouri et al. (2019) identified genistein, chrysin, galangin, rosmarinic acid, caffeic acid, and apigenin as the primary phytochemicals present in propolis. While, in the studies conducted by Ghavidel et al. (2021), the predominant phytochemical compounds in Tunisian propolis were found to be pinostrobin chalcone (22.90%), pinocembrin (6.14%), and benzothiadiazole (5.76%). Conversely, the ethanolic extract of Turkish propolis was reported to contain chrysin, phenyl ester of caffeic acid, pinocembrin, galangin, naringenin, kaempferol, trans‐cinnamic acid, caffeic acid, myristin, and quercetin (Bozkuş, Değer, and Yaşar 2021). Variations in the primary phytochemical compounds and their concentrations, as well as total phenolic content (TPC), total flavonoid content (TFC), and antioxidant activity of propolis extracts across different studies, can be attributed to factors such as geographical location, collection timing, and the type of propolis. Additionally, the extraction methods and conditions play a crucial role in these variations (Sambou et al. 2020).

**TABLE 1:** Phytochemicals identified in PE using the high‐performance liquid chromatography method.

No	RT (min)	Proposed compound	Area (%)	Reference/standard used
1	9.863	Galic acid	0.7	Standard
2	16.720	Caffeic acid	0.3	Gargouri et al. (2019)
3	26.603	Ferulic acid	4.1	Gargouri et al. (2019)
4	28.520	Isorhamnetin	0.2	Gargouri et al. (2019)
5	37.420	Pinobanksin	1.0	Gargouri et al. (2019)
6	44.787	Apigenin	18.9	Standard
7	46.983	Kaempferol	10.6	Gargouri et al. (2019)
8	51.337	Pinocembrin	5.9	Gargouri et al. (2019)
9	52.407	Genistein	1.8	Gargouri et al. (2019)
10	54.410	Chrysin	0.4	Gargouri et al. (2019)
11	55.263	CAPE	0.5	Gargouri et al. (2019)
12	55.543	Galangin	0.5	Gargouri et al. (2019)

Abbreviation: RT: retention time.

### 3.2 Chemical Composition of Probiotic Ice Creams

The chemical composition of the probiotic ice cream samples is presented in Table 2. The fat, protein, carbohydrate, and ash contents of these samples were found to range from 4.87% to 4.96%, 5.79% to 5.86%, 28.94% to 29.07%, and 0.92% to 0.97%, respectively. Furthermore, varying levels of PE did not significantly influence these chemical parameters. Consistent with these findings, Bengi et al. (2023) noted that the addition of different levels of PE did not significantly affect the chemical composition of Kefir, including its protein, fat, and ash content. Similarly, Demir Özer (2021) reported no significant impact of PE on the ash, fat, and protein levels in ice cream. However, the control ice cream exhibited a dry matter content of 40.72%, which increased with higher levels of extract powder, this increase was statistically significant only at the 0.8% extract level (*p* < 0.05). In support of these findings, Guler‐Akin, Goncu, and Akin (2016) demonstrated an increase in the dry matter content of probiotic yogurt following the addition of carob extract.

**TABLE 2:** Chemical composition of probiotic ice cream samples enriched with different levels of propolis extract (PE).

Treatments	Fat (%)	Protein (%)	Carbohydrate (%)	Ash (%)	Dry matter (%)
Control (0% PE)	4.95 ± 0.16^a^	5.86 ± 0.09^a^	28.98 ± 0.53^a^	0.93 ± 0.04^a^	40.72 ± 0.29^b^
0.2% PE	4.96 ± 0.13^a^	5.83 ± 0.11^a^	29.01 ± 0.44^a^	0.95 ± 0.02^a^	40.75 ± 0.38^ab^
0.4% PE	4.93 ± 0.21^a^	5.84 ± 0.05^a^	28.94 ± 0.51^a^	0.93 ± 0.02^a^	40.91 ± 0.23^ab^
0.6% PE	4.93 ± 0.15^a^	5.81 ± 0.12^a^	29.03 ± 0.39^a^	0.97 ± 0.06^a^	41.12 ± 0.17^ab^
0.8% PE	4.87 ± 0.18^a^	5.79 ± 0.09^a^	29.07 ± 0.62^a^	0.92 ± 0.05^a^	41.38 ± 0.33^a^

*Note:* Values represent mean (*n* = 3) ± SD. Different letters in each column represent significant difference at 5% level of probability among samples. Control: samples without propolis extract (PE) or (0% PE), 0.2% PE: samples containing 0.2 w/w PE, 0.4% PE: samples containing 0.4 w/w PE, 0.6% PE: samples containing 0.6 w/w PE, and 0.8% PE: samples containing 0.8 w/w PE.

### 3.3 Probiotic Viability in Ice Creams

**FIGURE 1:**
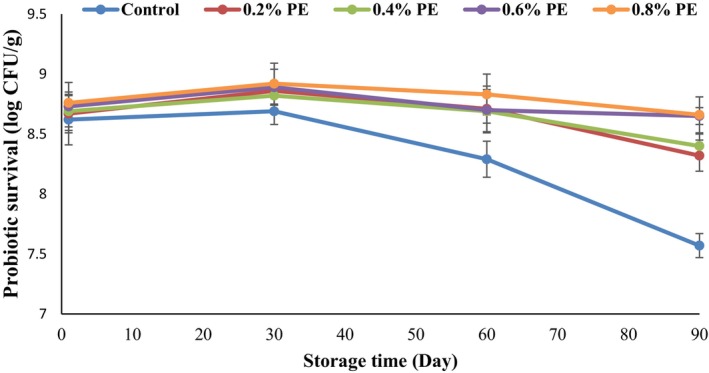
Survival of probiotic bacteria (log CFU/g) in ice cream samples enriched with different levels of propolis extract (PE). Control: Samples without PE or (0% PE), 0.2% PE: Samples containing 0.2 w/w PE, 0.4% PE: Samples containing 0.4 w/w PE, 0.6% PE: Samples containing 0.6 w/w PE, and 0.8% PE: Samples containing 0.8 w/w PE.

The findings from the investigation on the impact of varying levels of PE on the survival of *B. coagulans* probiotic bacteria during a 90‐day storage in the freezer are illustrated in Figure 1. Initially, the probiotic counts in the ice cream samples ranged from 8.62 to 8.76 log CFU/g, with no statistically significant differences (*p* > 0.05) detected among the various samples. Over the storage duration, a slight increase in the probiotic counts was followed by a gradual decline in the ice cream samples. A number of factors might influence the survival of probiotic bacteria, including the initial concentrations of the bacterial strain used, temperature, pH levels, the nature of the food carrier, and the conditions under which they are stored, as well as the processes of freezing and thawing (Mendonça et al. 2022; Putta et al. 2018). In contrast to the control samples, where the lowest level of *B. coagulans* (7.57 log CFU/g) was observed on day 90 of storage, the PE enriched ice creams exhibited enhanced viability of the probiotics bacteria (*p* < 0.05). The probiotic bacterial counts in the ice cream enriched with the PE ranged from 8.32 to 8.66 log CFU/g. According to research reports, the phenolic compounds present in extracts can serve as an energy source for probiotics, thereby enhancing their survival in the product (Martins et al. 2013). Rouhzadeh and Bahramian (2020) demonstrated that antioxidant compounds can absorb oxygen and eliminate it from the vicinity of microorganisms, potentially improving the viability of probiotics.

Generally, ice cream serves as an effective medium for delivering probiotic bacteria to consumers, as its pH is nearly neutral, thereby providing protection to the probiotics (Terpou et al. 2019). Despite the stressful conditions of freezing for probiotic bacteria, sugar in ice cream formulation plays the role of a cryoprotectant and improves the survival of probiotics (Ibrahim et al. 2022). Haghani et al. (2021) noted a gradual decrease in probiotic bacteria in ice cream throughout the storage period, suggesting that the inclusion of high concentrations of cranberry peel powder could enhance the viability of probiotics in this product. Similar to these reports, during our studies we observed a gradual decline in the quantity of probiotic bacteria in the ice cream samples during the storage period; although, the counts remained within the recommended threshold for probiotic dairy products (≥ 7 log CFU/g). This decrease is likely attributed to the presence of air within the ice cream matrix, as probiotics are sensitive to oxygen. Moreover, this reduction in the counts of *B. coagulans* can be partially attributed to the freezing process, which may inflict damage on bacterial cells (de Souza et al. 2019). According to previous reports, the cell membranes of probiotics could be damaged during the freezing process as a result of mechanical stresses caused by the formation of ice crystals in the surrounding medium (intercellular space) or within the cells themselves. The formation of smaller ice crystals can be reduced by utilizing rapid freezing techniques, which minimize the size of the ice crystals (Fowler and Toner 2006; Sun 2005; Terpou et al. 2019). Furthermore, the viability of probiotics may be further diminished during the thawing phase, as the cells encounter osmotic stresses (Jay, Loessner, and Golden 2008).

### 3.4 pH And Acidity of Probiotic Ice Creams

The pH level is known to have significant effects on the flavor perception of dairy products. Homayouni et al. (2008) indicated that typically ice cream has a pH range of approximately 6 to 7. While acidity refers to the amount of acid in the food samples. The optimal acidity of ice cream is 0.198% (Shamshad et al. 2023). The pH and acidity levels of various probiotic ice cream samples are presented in Table 3. During the initial storage period, the pH and acidity values of the ice cream samples ranged from 6.34% to 6.37% and 1.30% to 1.34% (calculated based on lactic acid contents), respectively. Furthermore, different concentrations of propolis extract (PE) had no significant influence on the pH and acidity of the probiotic ice cream during this time period. However, over time a gradual decline in pH was noted and an increase in acidity (*p* < 0.05), with final values reaching 6.17% to 6.29% and 1.47% to 1.68% on the last day of storage, respectively. The decline in pH values has been reported by Murtaza et al. (2004), who observed a gradual decline in the pH of ice cream from 6.85 to 6.78 during the storage period. This reduction in pH values can be attributed to the incorporation of various stabilizers and emulsifiers. Similar phenomenon showing a decrease in the pH values of probiotic ice creams during storage at freezer temperatures has also been reported by others (Ahmad et al. 2020; Salık 2019).

**TABLE 3:** The changes in pH and acidity of probiotic ice cream samples enriched with different levels of propolis extract (PE) during storage period.

Treatments	Storage time (Day)	pH	Acidity (% LA)
Control (0% PE)	1	6.37 ± 0.03^Aa^	1.30 ± 0.04^Da^
	30	6.25 ± 0.01^Bb^	1.37 ± 0.02^Ca^
	60	6.22 ± 0.02^Cb^	1.51 ± 0.02^Ba^
	90	6.17 ± 0.02^Db^	1.68 ± 0.03^Aa^
0.2% PE	1	6.37 ± 0.00^Aa^	1.31 ± 0.02^Da^
	30	6.34 ± 0.02^Ba^	1.37 ± 0.03^Ca^
	60	6.32 ± 0.00^Ba^	1.43 ± 0.01^Bb^
	90	6.29 ± 0.01^Ca^	1.54 ± 0.03^Ab^
0.4% PE	1	6.35 ± 0.02^Aa^	1.30 ± 0.02^Ca^
	30	6.33 ± 0.03^Aa^	1.33 ± 0.03^Ca^
	60	6.30 ± 0.03^ABa^	1.39 ± 0.03^Bb^
	90	6.28 ± 0.01^Ba^	1.48 ± 0.04^Abc^
0.6% PE	1	6.34 ± 0.03^Aa^	1.33 ± 0.01^Ca^
	30	6.33 ± 0.01^Aa^	1.35 ± 0.03^Ca^
	60	6.29 ± 0.03^ABa^	1.41 ± 0.02^Bb^
	90	6.27 ± 0.03^Aa^	1.48 ± 0.01^Ac^
0.8% PE	1	6.35 ± 0.03^Aa^	1.34 ± 0.03^Ca^
	30	6.32 ± 0.03^Aa^	1.37 ± 0.02^BCa^
	60	6.31 ± 0.01^Aa^	1.41 ± 0.02^Bb^
	90	6.26 ± 0.02^Ba^	1.47 ± 0.03^Ac^

*Note:* The values are expressed as mean ± standard deviation of triplicate. Large and small different letters indicate significant difference at 5% level of probability among times and samples (*p* > 0.05). Control: samples without propolis extract (PE) or (0% PE), 0.2% PE: samples containing 0.2 w/w PE, 0.4% PE: samples containing 0.4 w/w PE, 0.6% PE: samples containing 0.6 w/w PE, and 0.8% PE: samples containing 0.8 w/w PE.

In samples containing PE, the changes in pH and acidity during the storage period were less pronounced compared to the control sample. This reduction in pH and acidity changes in ice cream samples with varying concentrations of PE is likely attributed to the antimicrobial properties of the extract, and a reduction in fat oxidation due to its antioxidant effects. Elkassas, Yassin, and Taksira (2023) reported that the addition of propolis aqueous extract did not affect the pH of raw milk at the beginning of the storage period. According to their reports, while the pH values of milk and yogurt samples decreased over the storage period, the rate of decline in samples containing PE was lower than that of the control sample. Similarly, Santos et al. (2019) observed that the addition of red propolis extract had no significant effect on the pH values and acidity of probiotic yogurt during the storage period.

### 3.5 Apparent Viscosity of Probiotic Ice Creams

The findings regarding the apparent viscosity of probiotic ice cream samples are presented in Table 4. Viscosity is a measure of a substance's resistance to flow, typically expressed in centipoise (cP). Viscosity plays a crucial role in determining the quality of ice cream, with its level being influenced by the product's structure (Wang et al. 2013). Additionally, viscosity plays a significant role in determining the mouthfeel and flavor profile of ice cream. A rise in viscosity results in a denser texture that enhances the mouthfeel of the ice cream. During the study, the control ice cream samples exhibited an apparent viscosity of 29.11 cP, while, the samples containing PE showed higher viscosity with a gradual rise in the apparent viscosity with increasing PE concentrations. Notably, this increase was significant (*p* < 0.05) only in the sample containing the highest concentration of extract powder (30.17 cP, 0.8%) when compared to the control sample. This enhancement in viscosity can be attributed to the partial increase in the dry matter content of the samples due to the high level of extract incorporation. Compared to our results, higher viscosity values for probiotic ice cream has been reported by Hanafi, Kamaruding, and Shaharuddin (2022), who reported viscosity values of 32.37 cP. Conversely, Ibrahim et al. (2022) showed lower viscosity values of cP for probiotic ice cream. The differing trends observed in these studies may be linked to the characteristics of the additives and their respective incorporation levels in the product formulation. Butt, IjazAhmad, and Shahzadi (1999) suggested that the viscosity of ice cream is influenced by various stabilizers and emulsifiers in the ice cream. According to their reports, a noticeable reduction in ice cream viscosity during storage was seen resulting from the incorporation of different stabilizers and emulsifiers.

**TABLE 4:** Apparent viscosity, overrun, melting rate, color indices, hardness, and adhesiveness of probiotic ice cream samples enriched with different levels of propolis extract (PE).

Treatments	Apparent viscosity (cP)	Overrun (%)	Melting rate (%)	L*	a*	b*	ΔE	Hardness (*N*)	Adhesiveness (N.S)
Control (0% PE)	29.11 ± 0.46^b^	43.17 ± 1.85^a^	71.46 ± 1.93^a^	90.96 ± 1.13^a^	0.83 ± 0.05^a^	5.29 ± 0.14^d^	—	43.59 ± 1.17^b^	−2.16 ± 0.07^a^
0.2% PE	29.38 ± 0.41^ab^	42.65 ± 1.79^a^	71.63 ± 1.38^a^	89.15 ± 0.90^ab^	0.84 ± 0.02^a^	5.69 ± 0.17^c^	1.94 ± 0.19^d^	44.37 ± 1.35^ab^	−2.19 ± 0.03^a^
0.4% PE	29.59 ± 0.55^ab^	42.13 ± 2.04^a^	71.85 ± 1.84^a^	88.37 ± 0.84^bc^	0.86 ± 0.08^a^	5.87 ± 0.10^bc^	2.65 ± 0.26^c^	44.96 ± 1.24^ab^	−2.17 ± 0.06^a^
0.6% PE	29.84 ± 0.39^ab^	41.40 ± 1.56^a^	73.04 ± 1.55^a^	86.99 ± 1.17^cd^	0.86 ± 0.05^a^	6.14 ± 0.19^b^	4.06 ± 0.24^b^	45.73 ± 1.57^ab^	−2.24 ± 0.08^a^
0.8% PE	30.17 ± 0.28^a^	40.96 ± 1.37^a^	73.48 ± 1.47^a^	86.12 ± 0.89^d^	0.90 ± 0.07^a^	6.53 ± 0.15^a^	5.00 ± 0.31^a^	46.35 ± 1.06^a^	−2.25 ± 0.06^a^

*Note:* Values represent mean (*n* = 3) ± SD of triplicate. Different letters in each column represent significant difference at 5% level of probability among samples. Control: samples without propolis extract (PE) or (0% PE), 0.2% PE: samples containing 0.2 w/w PE, 0.4% PE: samples containing 0.4 w/w PE, 0.6% PE: samples containing 0.6 w/w PE, and 0.8% PE: samples containing 0.8 w/w PE.

### 3.6 Overrun of Probiotic Ice Creams

The overrun percentage of the probiotic and propolis incorporated ice cream samples is presented in Table 4. During the production of ice cream, the process of stirring incorporates air into the mixture, resulting in an increase in volume and expansion during freezing known as overrun. High‐quality ice creams are characterized by an appropriate percentage of overrun (Aloğlu, Gökgöz, and Bayraktar 2018). A low overrun percentage can lead to a texture that is excessively stiff, while an overly high overrun can produce a granular consistency. Generally, an overrun value between 15% and 50% is considered optimal for ice creams (Mehmetoğlu and Tarakçı 2023). The formulation of the ice cream, the methods of production, and the equipment utilized are all critical factors that influence the level of overrun in the final product. In the present study, the overrun levels of the ice cream samples analyzed ranged from 40.96% to 43.17%, which falls within the acceptable limits. Similar to our findings, Atsan and Çağlar (2008) reported lower overrun values ranging from 31.13% to 41.71% in their tested ice creams, while, Shazly et al. (2022) reported overrun values of 33.89% to 41.76%, for the probiotic ice creams. However, the overrun percentage for probiotic ice cream in Haghani et al. (2021) was notably higher at 56.56%. The discrepancies among the studies are believed to stem from differences in ice cream formulations, production methods, and the efficiency of the freezers used. Intriguingly, during analysis, the control sample exhibited the highest overrun, while an increase in the level of PE corresponded with a gradual, albeit negligible, decrease in the overrun of the ice cream samples. Converse to these findings, Mehmetoğlu and Tarakçı (2023) reported a statistical difference in the ice cream samples depending on the propolis concentrations (*p* < 0.05). They observed increasing volume index (overrun) with increasing propolis concentrations, which became insignificantly different on last day of storage (60 days). Conversely, Haghani et al. (2021) documented a reduction in the overrun percentage of probiotic ice cream samples that were enhanced with cranberry peel powder. In our studies, an increase in viscosity was observed with increasing concentrations of PE, and alternatively insignificant decrease in the overrun values was observed. Erkaya, Dağdemir, and Şengül (2012) had reported that with the increase in the viscosity of ice cream, the amount of air interred into the product structure decreases which might lead to decrease in the percentage of overrun and vice versa.

### 3.7 Melting Rate of Probiotic Ice Creams

The melting rates of probiotic ice cream samples are shown in Table 4. Meltdown represents a significant characteristic of frozen dairy products. It is affected by several factors, including the volume of air incorporated, the size of ice crystals, and the arrangement of fat globules (Muse and Hartel 2004). High‐quality ice creams exhibit a reduced meltdown rate, whereas those that melt quickly demonstrate a lower resistance to heat shock. The slower melting observed in ice cream with a higher overrun is primarily due to diminished heat transfer through the air bubbles (Alizadeh, Azizi‐Lalabadi, and Kheirouri 2014). In the present study, increasing concentrations of PE had no significant effect on the melting rate of ice creams and the melting rate values of ice cream samples were in the range of 71.465%–73.48%. Our obtained melting rate values are higher than the values obtained by Nouri and Entezari Sareshkeh (2022), while they are in the range reported by Hanafi, Kamaruding, and Shaharuddin (2022). Not only melting rate depends on the fat structure and rheological behavior of ice cream samples, but ice creams with lower viscosity and less overrun shows higher melting rate, because the air cells act as insulators in the ice cream (Akalın et al. 2018). The influence of fat content and overrun percentage on the melting rate of ice cream is well established. Hence, these statements could justify why varying levels of PE did not significantly affect the melting rate of our tested ice cream samples. Specifically, the addition of different concentrations of this extract did not lead to notable changes in either the fat content or the overrun of the samples when compared to the control. In agreement with these findings, Mehmetoğlu and Tarakçı (2023) also observed that different propolis concentrations in the ice cream samples did not make a statistically significant difference in the melting rate (*p* > 0.05). While, in contrast Haghani et al. (2021) reported a significant decrease in melting rate of probiotic ice creams after incorporation of cranberry peel powder. In general, this difference in the findings is probably related to the difference in the nature of the additives used and the ingredients of the ice cream formulation.

### 3.8 TPC and Antioxidant Activity of Probiotic Ice Creams

**FIGURE 2:**
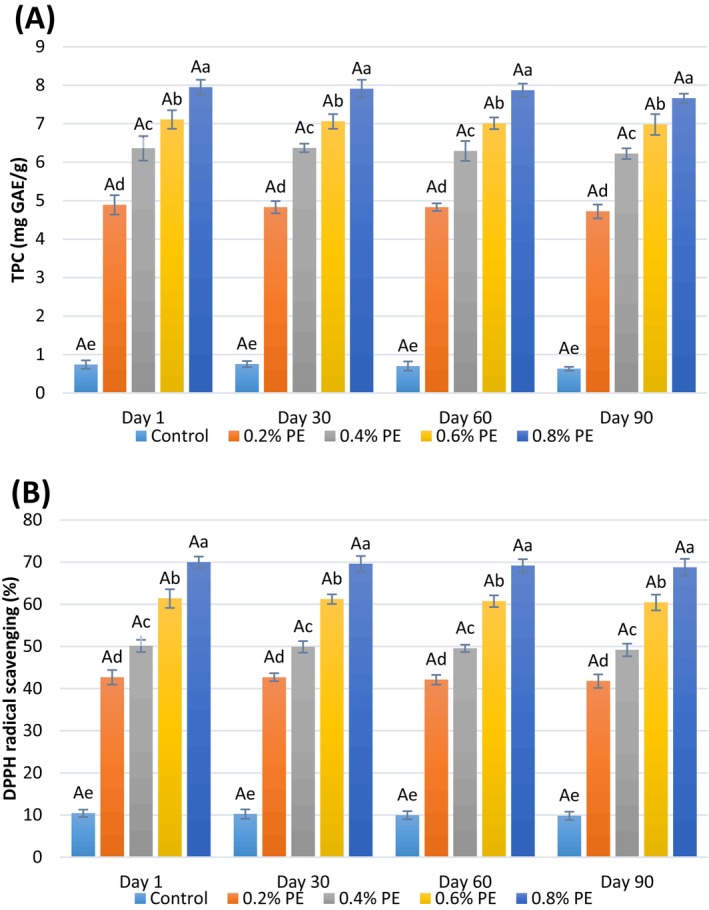
(A) TPC (mg GAE/g) and (B) DPPH radical scavenging (%) of probiotic ice cream samples enriched with different levels of propolis extract (PE). Bars represent mean (*n* = 3) ± SD of triplicate. Large and small different letters on the bars indicate significant difference at 5% level of probability among times and samples TPC: Total phenolic compounds, control: Samples without propolis extract (PE) or (0% PE), 0.2% PE: Samples containing 0.2 w/w PE, 0.4% PE: Samples containing 0.4 w/w PE, 0.6% PE: Samples containing 0.6 w/w PE, and 0.8% PE: Samples containing 0.8 w/w PE.

The total phenolic content (TPC) of probiotic ice cream samples was assessed using the Folin‐Ciocalteau method, while their antioxidant activity was evaluated through the DPPH radical inhibition test, over the storage period, with results illustrated in Figure 2. As anticipated, the control probiotic ice cream exhibited the lowest TPC at 0.74 mg GAE/g and an antioxidant activity of 10.43%. It has been noted that phenolic compounds are present in milk, which is attributed to their production during the catabolism of amino acids. Additionally, the pasteurization process can generate phenolic compounds through the Maillard reaction (Berktas and Cam 2021). A considerable portion of the antioxidant activity observed in the control ice cream sample can be attributed to the inclusion of milk and milk powder in its formulation, as whey proteins and casein are recognized as natural antioxidants (Khan et al. 2019). Upon the incorporation of propolis extract (PE) and the subsequent increase in its concentration from 0.2% to 0.8% in the ice cream formulation, both TPC and antioxidant activity showed a significant increase (*p* < 0.05), with TPC rising from 4.89 to 7.95 mg GAE/g and DPPH radical inhibition increasing from 42.68% to 69.96%. Throughout the storage period, a slight decline in TPC and antioxidant activity of the ice cream samples was noted; however, these variations were not statistically significant. The notable antioxidant activity of PE is attributed to its high levels of phenolic and flavonoid compounds. Studies have indicated that PE is resilient to processing conditions, such as temperature, and can retain its antioxidant properties in dairy products (Cottica et al. 2015). Furthermore, an increase in TPC and antioxidant activity of raw milk following elevated PE concentrations has also been reported in the research of Ghannadiasl and Jahdoust (2024). Gunes et al. (2024) conducted a study to evaluate the effectiveness of powdered propolis as a natural preservative in ice cream, using it as a model food. Their findings indicated that the total phenolic content of the propolis was 46.26 ± 1.18 mg GAE/mL. This variation may be attributed to the type of propolis utilized in the research.

### 3.9 Color of Probiotic Ice Creams

The color of the probiotic ice cream samples was analyzed using a calorimeter, with the resulting color indices presented in Table 4. The control sample exhibited the highest L* index and the lowest b* index, measuring 90.96 and 5.29, respectively. As the concentration of PE in the ice cream formulation increased, there was a gradual decrease in brightness and a corresponding increase in yellowness (*p* < 0.05). The sample containing 0.8% extract recorded the lowest L* index and the highest b* index, at 86.12 and 6.53, respectively. Notably, varying concentrations of the extract did not significantly affect the a* index, which remained within the range of 0.83 to 0.90. The observed darker and yellower hues in the ice creams can be attributed to the yellow pigmentation of the extract, as the color of dairy products is significantly influenced by the ingredients used in their formulation. The results indicate that with increasing PE concentration, the Δ*E* index also rose significantly (*p* < 0.05). In a study by Bengi et al. (2023), it was noted that the addition of PE did not significantly impact the L* and a* indices of strawberry‐flavored kefir, with only the b* index showing a significant increase as PE concentration rose. Zor and Sengul (2022) had reported a decline in the L* index alongside increases in the a* and b* indices of ice creams following enrichment with *Rosa pimpinellifolia* L. extracts. According to the statements of Mehmetoğlu and Tarakçı (2023), propolis is a naturally occurring resinous substance characterized by a unique and aromatic fragrance that can have color variations significantly based on their source and stage of maturity.

### 3.10 Texture of Probiotic Ice Creams

Texture is one of the important quality parameters of food products. Ice cream texture is generally influenced by overrun content, rheological properties, fat destabilization, ice crystal size, and ice phase volume (Muse and Hartel 2004; Segueni et al. 2023). The findings regarding the hardness and adhesiveness of the probiotic ice cream samples are given in Table 4. Our findings indicate that the hardness of the control sample measured 43.59 N, and as the concentration of PE powder increased, there was a gradual rise in hardness. However, this increase was only statistically significant at the 0.8% concentration level, where the hardness reached 46.35 N (*p* < 0.05). The notable increase in hardness of the ice creams after the addition of 0.8% PE (46.35 N) is likely associated with an increase in dry matter and viscosity, along with a slight reduction in the overrun percentage. Furthermore, varying concentrations of PE did not significantly affect the adhesiveness of the probiotic ice creams, with adhesiveness values ranging from −2.16 to −2.25. Mehmetoğlu and Tarakçı (2023) found that the addition of propolis powder to ice cream formulations at different levels did not significantly affect the hardness and stickiness values of the products. However, hardness values significantly differed after the 3rd day of storage compared to 30 and 60 days. The highest hardness value was found in the ICM1 sample (containing of 0.1% PE) stored for 60 days, while the lowest was in the control group. The ICM3 sample (with 0.3% PE) had the highest stickiness, while the control group had the lowest. After 30 days of storage, hardness values increased significantly (*p* < 0.05). The changes between concentrations and hardness values after 60 days were not statistically significant.

### 3.11 Sensory Evaluation of Probiotic Ice Cream

**FIGURE 3:**
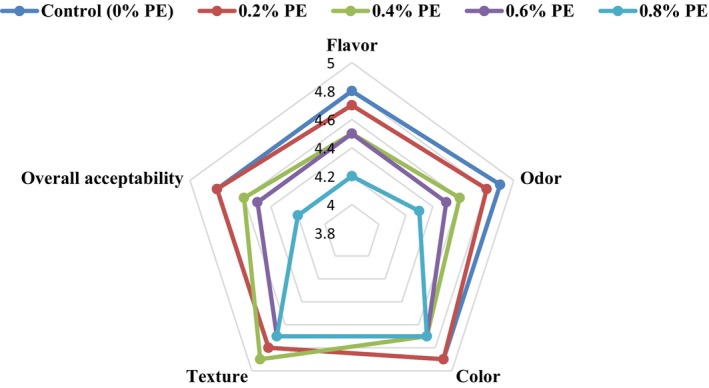
Sensory properties of probiotic ice cream samples enriched with different levels of propolis extract (PE). Control: Samples without PE or (0% PE), 0.2% PE: Samples containing 0.2 w/w PE, 0.4% PE: Samples containing 0.4 w/w PE, 0.6% PE: Samples containing 0.6 w/w PE, and 0.8% PE: Samples containing 0.8 w/w PE.

Sensory characteristics of probiotic ice cream samples including flavor, odor, color, texture, and overall acceptability are compared in Figure 3. As can be seen, the control sample had high sensory scores. Adding different concentrations of PE did not show a significant effect on the score of color and texture of ice cream, and increasing the concentration of the extract up to 0.6% did not have a significant effect on the flavor and overall acceptance of ice cream, and only the concentration at level 0.8% caused a significant decrease in the scores of flavor, odor and overall acceptability of the enriched ice creams compared to the control sample. However, all the samples examined in this research scored well and had good acceptance. In agreement with these results, Mehmetoğlu and Tarakçı (2023) found that color‐appearance scores during storage were insignificant, but the effect of different propolis concentrations was statistically significant. The control group had the highest scores, while the ICM5 (0.5% propolis added) group had the lowest. The structure‐texture scores were insignificant, with the control group having the highest. Taste–aroma scores were significantly affected by propolis concentration and storage time. Overall acceptability scores were significantly affected by propolis concentration. The control group was the most liked, and propolis addition negatively affected all sensory properties of ice creams. In another study conducted by Demir Özer (2021), ice cream samples treated with different propolis concentrations of 400, 800, and 1600 mg/L were assessed for their effectiveness against Listeria. Their findings from the sensory evaluation indicated that the control sample received the highest ratings across all sensory attributes. The sample with 400 mg/L of propolis (Group 1P) followed closely behind the control in scoring. Notably, the sample with the lowest concentration of propolis was the most favored among participants. As the concentration of propolis increased, the scores for appearance, taste, and aroma of the samples diminished. This decline is primarily attributed to the presence of phenolic acids and other volatile compounds in propolis, which impart a strong and distinctive flavor and aroma.

## 4 Conclusion

This study involved the formulation of a functional ice cream product incorporating *B. coagulans* and varying concentrations of propolis extract (PE). The physicochemical, sensory, and bioactive characteristics of the product were assessed throughout the storage period. Findings of the study indicated ice cream as a suitable delivery vehicle for the creation of probiotic‐enriched functional foods. While, propolis well‐recognized for its extensive health benefits and functional properties, showed a significant enhancement in total phenolic content (TPC) and enhanced antioxidant activity, all while maintaining the physicochemical and sensory quality of the prepared ice cream. Interestingly, the used PE concentrations in study contributed to the improved survival of probiotic bacteria. The ice cream developed with propolis and *B. coagulans* probiotic exhibited highly promising attributes, suggesting its potential as a novel functional food suitable for consumers of all age groups. In line with these results, further studies should be carried out to evaluate the effect of different types and higher concentrations of propolis extracts in different food matrix so as to develop novel functional foods with enhanced health benefits and better texture properties like melting rate. Consequently, the use of *B. coagulans* may represent a promising preventive and/or therapeutic strategy for addressing human diseases.

## Author Contributions

**Mehri Soodbar:** formal analysis (equal), investigation (equal), methodology (equal), writing – original draft (equal). **Naheed Mojgani:** data curation (equal), investigation (equal), supervision (equal), validation (equal), writing – review and editing (equal). **Mohammad Reza Sanjabi:** data curation (equal), investigation (equal), validation (equal), writing – review and editing (equal). **Saeed Mirdamadi:** data curation (equal), investigation (equal), validation (equal). **Mostafa Soltani:** data curation (equal), investigation (equal), validation (equal).

## Conflicts of Interest

The authors declare no conflicts of interest.

## Data Availability

Available data will be expressed on request.
